# Lung cancer patients with nephropathy as the first manifestation: Literature review and clinical study report

**DOI:** 10.3389/fonc.2022.1002155

**Published:** 2022-09-29

**Authors:** Qianqian Xu, Guming Zou, Li Zhuo, Hongmei Gao, Wenge Li

**Affiliations:** Department of Nephrology, China-Japan Friendship Hospital, Beijing, China

**Keywords:** lung cancer, membranous nephropathy, anti-phospholipase A2 receptor antibody, thrombospondin type-1 domain-containing 7A, NELL-1

## Abstract

**Background:**

To investigate the relationship between membranous nephropathy (MN) and lung cancer.

**Methods:**

To report patients with lung cancer detected by follow-up after the diagnosis of MN by renal biopsy in China-Japan Friendship Hospital from January 2010 to December 2019, and to study the prognosis of lung cancer-associated MN and have a review of the literature.

**Results:**

Lung cancer was detected in six patients followed for 1–27 months (median 8 months) after the diagnosis of MN: including four cases of lung adenocarcinoma, one case of carcinoma *in situ*, and one case of small cell lung cancer with multiple metastases. Five cases were in remission after surgical resection, and one case was remitted after chemotherapy. Six patients were negative for serum anti-PLA2R antibodies, and glomerular IgG subclass deposition detected by immunofluorescence was positive for IgG1 and IgG2. Glomerular PLA2R, THSD7A, and NELL-1 stainings were assessed in all six patients; one patient was positive for glomerular PLA2R staining, two patients were positive for glomerular THSD7A staining, and all patients were negative for NELL-1 staining. A literature review of the relationship between MN and lung cancer was performed: seven articles about cancer-associated MN were searched, reporting 32 cases of MN associated with lung cancer, among which 14 cases had nephropathy as the first manifestation and only five patients had remission of MN after treatment of lung cancer.

**Conclusions:**

A few lung cancer patients have nephropathy as the first clinical manifestation, and MN can also be remitted after treatment of lung cancer.

## Introduction

Membranous nephropathy (MN) is a common pathological type leading to nephrotic syndrome (NS) in adults, accounting for 9.83% to 30% of primary glomerulonephritis (1, 2). MN is a pathologic entity characterized by the formation of immune complexes under the epithelial cells and diffuse thickening of the glomerular basement membrane observed by light microscopy (3). However, 75% of MN cases are idiopathic, whereas the remainder are associated with infections, malignancies, autoimmune diseases, and drug toxicity (4).

In recent years, there have been more reports of malignancy-associated MN. In this paper, we summarized and analyzed the literature of cancer-associated MN in the past 20 years and searched seven domestic and foreign articles, totaling 113 patients with cancer-associated MN. Lung cancer was the most common type of tumor, accounting for 32 cases (28.3%), and among them, nephropathy was the first manifestation in 14 cases (12.4%). We also reported six cases of lung cancer patients who were followed up after the diagnosis of MN by kidney biopsy in China-Japan Friendship Hospital from January 2010 to December 2019 and analyzed their clinical characteristics and prognosis to explore the relationship between lung cancer and MN.

## Materials and methods

### Data sources and searches

We searched literature about cancer-associated MN in the past 20 years, and a total of seven articles were selected to summarize the relationship between lung cancer and MN. We also reported the clinical data and pathological characteristics of patients who were followed up and found to have lung cancer after diagnosis of MN by renal biopsy in China-Japan Friendship Hospital from January 2010 to December 2019.

This study was approved by the Ethics Committee of China-Japan Friendship Hospital (approval number: 2019-17-K12).

### Patient selection

The inclusion criteria for patients with lung cancer-associated MN were as follows: (1) renal histological and immunopathological changes consistent with secondary membranous nephropathy (SMN) (5); (2) lung cancer was detected at the same time or within several years after the diagnosis of MN, and MN was in remission after treatment of lung cancer, or MN recurred when lung cancer recurred; (3) secondary causes such as systemic lupus erythematosus, hepatitis B, and hepatitis C infection were excluded, as well as no use of non-steroidal anti-inflammatory drugs, gold agents, penicillamine, and other drugs and no history of exposure to organic solvents and mercury.

### Clinical and biological data

Patients with lung cancer-associated MN were counted for the following aspects of clinical data: (1) age, gender ratio, 24-h urine protein quantification, serum albumin, blood creatinine, eGFR, and anti-PLA2R antibodies; (2) the main clinical manifestations of the patients, time of diagnosis of MN and lung cancer; (3) type and treatment of lung cancer, the prognosis of lung cancer, and remission of MN after treatment of lung cancer.

The estimated glomerular filtration rate (eGFR) was estimated using the EPI formula (6).

### Analysis of renal biopsies

Percutaneous renal puncture was performed, and kidney tissue specimens were obtained in three parts in all cases for light microscopy, immunofluorescence, and electron microscopy. (1) Light microscopy: light microscopic specimens all contained more than 10 glomeruli, were paraffin-embedded, sectioned 2 μm thick, and stained with HE, PAS, PASM, and MASSON, respectively. (2) Immunofluorescence: Frozen sections were used to detect IgG, IgA, IgM, C3, C1q, FRA, and IgG subclasses (IgG1, IgG2, IgG3, and IgG4) by direct immunofluorescence. (3) Electron microscopy: All kidney biopsy specimens were sent to the electron microscopy laboratory of China-Japan Friendship Hospital for examination. Renal pathological typing and diagnosis were referred to our pathological standards for renal biopsies.

### Study reagents

Serum anti-PLA2R antibody assay: Patients after August 2016 were tested using the human anti-phospholipase A2 receptor antibody enzyme immunoassay kit (Shanghai Lianshuo Biological), and each step was performed according to the instructions, and finally the expression of serum anti-PLA2R to be tested was calculated according to the standard curve. Serum anti-PLA2R antibody ≥20 IU/ml was considered positive.

Paraffin-embedded sections of formalin-fixed renal tissue were utilized for immunohistochemistry (IHC) using rabbit polyclonal anti-PLA2R (1:500; Sigma-Aldrich, Germany), rabbit polyclonal anti-THSD7A (1:400; Sigma-Aldrich), and rabbit polyclonal anti–NELL-1 antibody (1:800; Sigma-Aldrich) as the primary antibodies. The glomerular expressions of PLA2R, THSD7A, and NELL-1 were detected according to the same protocol as reported previously.

## Results

### Literature review

We searched and summarized the literature on cancer-associated MN from 2000 to the present, and seven domestic and foreign articles were selected (7–13), totaling 113 patients, and the most common tumor was lung cancer in 32 cases (28.3%). Among the lung cancer patients, 14 cases (12.4%) had nephropathy as the first manifestation; a summary of the literature on cancer-associated MN is shown in **Table 1**. Among them, 12 patients with detailed information of the type of lung cancer, treatment and prognosis of lung cancer, and remission of MN are shown in **Table 2**.

**Table 1:** Summary of the literature on lung cancer-associated MN.

	Li et al. (7)	Bjørneklett et al. (8)	Qu et al. (9)	Zhang et al. (10)	Ohtani et al. (11)	Lönnbro-Widgren et al. (12)	Lefaucheur et al. (13)	Total
Follow-up period	2005-2008	1988-2003	1997-2009	2001-2017	1985-2002	2000-2012	1994-2001	
Mean age, years	53.6 ± 6.7	65 ± 11	64.4 ± 8.7	63.4 ± 7.9	64 (54, 80)	68 ± 10	73 (65, 78)	
Total number, n	10	33	8	12	10	16	24	113
Patients with lung cancer, n (%)	5 (50)	6 (18.2)	3 (37.5)	5 (41.7)	2 (20)	3 (18.7)	8 (33.3)	32 (28.3)
Diagnosed with cancer before the diagnosis of MN, n (%)	1 (10)	NA	3 (37.5)	0	0	0	NA	4 (3.5)
Diagnosed with cancer at the same time or following the diagnosis of MN, n (%)	4 (40)	NA	0	5 (41.7)	2 (20)	3 (18.7)	NA	14 (12.4)
Remission of MN after treatment of lung cancer, n (%)	1 (10)	NA	NA	2 (16.7)	0	1 (6.2)	4 (16.7)	8 (7.1)
Number of deaths during lung cancer follow-up, n (%)	1 (10)	NA	NA	2 (16.7)	2(20)	2 (12.5)	NA	7 (6.2)

NA, Not available.

**Table 2:** Review of the literature on lung cancer patients with nephropathy as the first manifestation.

Case no.	Pathology and markers of MN			Histology of lung cancer	Treatment of lung cancer		Remission of lung cancer	Remission of MN
	Pathological type	Glomerular PLA2R	PLA2R-Ab (RU/ml)		S	C/R		
1 (7)	Secondary MN	NA	+	Squamous cell carcinoma	S	C	Relapse	NR
2 (7)	Secondary MN	NA	–	Adenocarcinoma		C	No	NR
3 (7)	Secondary MN	NA	–	Squamous cell carcinoma	S	C	Yes	CR
4 (7)	Secondary MN	NA	–	Squamous cell carcinoma		C	No	NR
5 (10)	Secondary MN	–	–	Adenocarcinoma	S		Yes	PR
6 (10)	Secondary MN	–	–	Adenocarcinoma	S	C	No	NR
7 (10)	Secondary MN	–	–	Carcinoma *in situ*	S		Yes	CR
8 (10)	Secondary MN	–	–	Squamous cell carcinoma	S	C	No	NR
9 (10)	Secondary MN	+	+	Carcinoma *in situ*	S		Yes	PR
10 (12)	Secondary MN	–	NA	Lung cancer	S	R	No	NR
11 (12)	Secondary MN	–	NA	Lung cancer	No		No	NR
12 (12)	Secondary MN	–	NA	Lung cancer	S		Yes	CR

R, radiotherapy; S, surgery; C, chemotherapy; CR, complete remission; NR, no remission; PR, partial remission; NA, Not available.

As seen in **Table 2**, all patients with lung cancer-associated MN had pathology characterized by secondary membranous nephropathy, and only two patients were positive for serum anti-PLA2R antibody. Six patients with unremitting lung cancer had unremitting MN as well, one relapsed, and five patients had remission of MN after treatment and remission of lung cancer. However, the specifics of remission of MN after lung cancer treatment were not documented in these patients.

### Clinical manifestations of lung cancer patients with nephropathy as the first manifestation in our center

In the follow-up of our center, we found that the pathology of six lung cancer patients with nephropathy as the first manifestation was secondary MN, and the patients were found to have lung cancer at 1–27 months (median 8 months) after the diagnosis of MN. Among them, there were three men and 3 women, the age of onset was 47–70 years, and the median age was 63 years. The histology of lung cancer included four cases of adenocarcinoma, one case of carcinoma *in situ*, and one case of small cell lung cancer with multiple metastases. Case 1 was found to have an occupying lesion in the lower left lung by chest CT without respiratory symptoms 27 months after diagnosis of MN. Adenocarcinoma was diagnosed through puncture pathology, and surgery was performed. Case 2 developed edema of both lower extremities without obvious incentives; chest CT was performed, and pulmonary nodules were found at the same time of diagnosis of MN. One month later, puncture pathology showed adenocarcinoma of the right lower lobe of the lung, and surgery was performed. Case 3 was mainly manifested with edema of both lower extremities and had no respiratory symptoms. One month after the diagnosis of MN, a mass in the lower lobe of the left lung was found during chest CT examination. The puncture pathology showed adenocarcinoma, and surgery was performed. Case 4 was found to have an occupying lesion in the left lower lobe of the lung 7 months after diagnosis of MN without respiratory symptoms. The puncture pathology showed adenocarcinoma of the lung, and surgery was performed. Case 5 was admitted to the hospital with increased foamy urine and was found to have a right lower lung nodule by chest CT 9 months after the diagnosis of MN, and the pathology showed carcinoma *in situ*, which was treated surgically. Case 6 was admitted to the hospital due to edema of both lower extremities, and after 13 months of diagnosis of MN, he consulted the respiratory department due to “cough and hemoptysis for 2 months”. Chest CT showed central lung cancer in the upper lobe of the left lung with pulmonary atelectasis and puncture pathology, and PET-CT showed small cell lung cancer with multiple metastases, which were inoperable and treated with chemotherapy. The detailed information of the six patients are shown in **Table 3**.

**Table 3:** Clinical and pathological features of lung cancer patients with nephropathy as the first manifestation.

Case no.	Pathology and markers of MN					Histology of lung cancer	Treatment of lung cancer		Remission of lung cancer	Remission of MN
	Pathological type	PLA2R-Ab (RU/ml)	Glomerular PLA2R	Glomerular NELL-1	Glomerular THSD7A		S	C/R		
1	Secondary MN	–	–	–	–	Infiltrative adenocarcinoma	S		Yes	CR
2	Secondary MN	–	–	–	–	Infiltrative adenocarcinoma	S	C	Yes	CR
3	Secondary MN	–	–	–	–	Infiltrative adenocarcinoma	S		Yes	PR
4	Secondary MN	–	–	–	–	Mucinous adenocarcinoma	S		Yes	CR
5	Secondary MN	–	+	–	+	Carcinoma *in situ*	S		Yes	CR
6	Secondary MN	–	–	–	+	Small cell lung cancer with multiple metastases		C	Yes	CR

R, radiotherapy; S, surgery; C, chemotherapy; CR, complete remission; NR, no remission; PR, partial remission.

All six patients had “bilateral lower limb edema” as the main manifestation, and five patients had massive proteinuria, showing nephrotic syndrome. One patient had mildly elevated blood creatinine at the beginning of the disease, and serum anti-PLA2R antibody was negative in six patients.

### Renal pathology

#### Light microscopy

Glomerular basement membrane thickening, mild mesangial cell hyperplasia, and mesangial matrix increases were seen in all patients under light microscope, and no glomerular segmental loop necrosis, crescent, and other lesions were found. Two patients had mild interstitial fibrosis, and one had ischemic injury.

#### Electron microscopy

All patients had electron-dense deposits on the epithelial side, four patients had a small amount of electron dense deposition in the mesangial area, and two patients had a small amount of electron dense deposition in the subendothelial area.

#### Immunofluorescence

Five patients showed full bright features (IgG, IgM, IgA, C3, C1q, and FRA were deposited), and one patient had IgG, IgM, C3, and FRA deposited. Glomerular IgG subclass deposition was detected by immunofluorescence: six patients were positive for IgG1 and IgG2, only one case was positive for IgG3, and three cases were positive for IgG4 (**Table 4**).

**Table 4:** Pathological and immunofluorescent features of lung cancer patients with nephropathy as the first manifestation.

Case no.	Mesangial proliferation	IgG	IgM	IgA	C3	C1q	IgG1	IgG2	IgG3	IgG4
1	1+	3+	2+	2+	2+	2+	3+	2+	0	0
2	2+	3+	2+	2+	3+	2+	3+	2+	0	2+
3	3+	4+	1+	2+	2+	2+	3+	2+	0	2+
4	2+	3+	2+	2+	2+	2+	3+	2+	0	3+
5	1+	2+	1+	0	3+	0	2+	1+	1+	0
6	2+	3+	2+	2+	2+	2+	3+	2+	0	0

#### Immunohistochemistry

**Figure 1:**
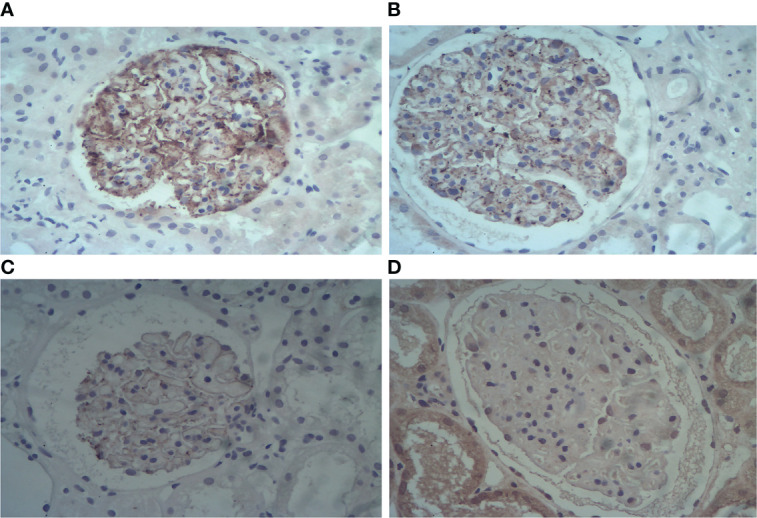
Representative images of positive glomerular PLA2R staining in case 5 **(A)** and positive THSD7A staining in case 5 **(B)** and case 6 **(C)** as determined by immunohistochemistry. Negative glomerular NELL-1 staining in case 2 **(D)**. (Immunohistochemistry staining, magnification ×400).

Glomerular PLA2R, THSD7A, and NELL-1 staining was performed on formalin-fixed, paraffin-embedded (FFPE) sections using immunohistochemistry (IHC) procedure as previously described. Subepithelial granular pattern staining was considered positive, while faintly appreciable staining of the tubular brush border and along the capillary walls was considered negative (shown in **Figure 1**).

#### Treatment and prognosis

Six patients were found to have lung cancer 27, 1, 1, 7, 9, and 13 months after the diagnosis of MN. All patients had remission of MN after surgical resection of tumor or chemotherapy, and there was no significant change in renal function before and after treatment. The manifestations of MN before and after lung cancer treatment are shown in **Table 5**.

**Table 5:** Prognosis of lung cancer patients with nephropathy as the first manifestation.

Case no.	Gender	Age	Time from biopsy to identification of malignancy (months)	Manifestation of MN before lung cancer treatment			Manifestation of MN after lung cancer treatment		
				Proteinuria (g/day)	ALB (g/L)	Scr (μmol/L)	Proteinuria (g/day)	ALB (g/L)	Scr (μmol/L)
1	M	66	27	6.62	30.5	65.2	0.52	35.2	87.5
2	M	63	1	8.37	28.4	119.3	0.32	39.4	109.4
3	F	70	1	8.81	22.6	61.9	1.52	32.5	77.6
4	F	58	7	4.85	30.4	58.3	0.58	34.6	56.4
5	F	47	9	2.7	36.2	44.6	0.24	38.6	45.9
6	M	63	13	14.67	24.5	65.4	0.28	35.3	59.1

## Discussion

Since Lee et al. (14) first proposed the existence of a relationship between NS and malignancy in 1996, the relationship between nephropathy and malignancy has been widely concerned by clinicians. In clinical practice, cancer-associated nephropathy is on the rise, mostly with proteinuria or NS as the main first symptom (8). The malignant tumors that cause kidney disease are mainly divided into two categories: solid tumors and non-solid tumors. Solid tumors include lung cancer, colon cancer, and renal cell carcinoma. Non-solid tumors include non-Hodgkin’s lymphoma and multiple myeloma. Among them, MN is more closely related to solid tumors. Clinically, the pathological types of cancer-associated nephropathy are not typical and the pathogenesis is not clear.

Napat et al. (15) performed a meta-analysis on the incidence and characteristics of cancer and MN and showed that the incidence of cancer-associated MN was around 10% and the mean age of cancer in patients with MN was (67 ± 7) years, with lung cancer being the most common, followed by gastric, intestinal, prostate, and breast cancers. We summarized all the seven articles on “cancer-associated MN” from 2000 to the present: there were 113 patients, 78 men, and the most common was lung cancer in 32 cases (18 adenocarcinoma, 12 squamous cell carcinoma, and 2 *in situ* carcinoma), accounting for 28.3%. Among them, 14 cases (12.4%) had nephropathy as the first manifestation. Six patients with unremitting lung cancer had unremitting MN as well, one relapsed, and five patients had remission of MN after treatment and remission of lung cancer.

Our follow-up analysis of six cases of lung cancer patients with nephropathy as the first manifestation found in China-Japan Friendship Hospital in the past 10 years revealed that the median age was 63 years, NS was the main manifestation, and all renal pathology showed features of secondary MN, with clinical exclusion of MN due to secondary causes such as hepatitis B, hepatitis C infection, and SLE. Among them, there were four cases of lung adenocarcinoma, one case of carcinoma *in situ*, and one case of small cell lung cancer with multiple metastases. Moreover, most of these patients had bilateral lower limb edema as the main manifestation at the time of discovery of MN, without clinical manifestations related to lung cancer and elevated specific tumor markers. Therefore, for elderly patients with MN, we cannot rely solely on clinical manifestations and positive tumor markers to rank for the presence of cancer, and routine chest CT is required to screen for lung cancer if necessary. During the follow-up of elderly patients with MN, it is necessary to pay close attention to the changes of the disease, and for patients who are not in remission with conventional treatment, regular systemic examinations should be performed to exclude the presence of tumors.

In the present study, the pathological manifestation of lung cancer-associated MN was mainly characterized by secondary MN, consistent with previous reports (9). Further analysis of IgG subclass in the renal tissue in our patients revealed that IgG subclasses were predominantly IgG1 and IgG2 deposition. It has been shown that IgG4 deficiency and IgG1 and IgG2 deposition in renal biopsy tissues are associated with malignancy-associated MN (11, 12). Ohtani et al.’s study also found that the glomerular IgG subclass deposition of cancer-associated MN was dominated by IgG1 and IgG2, which was different from the glomerular IgG4 deposition of IMN (11).

In 2009, Beck et al. (16) first detected M-type PLA2R antibodies in the blood circulation of patients with IMN, which are present in the serum of approximately 70%–80% of patients with IMN and are strongly associated with disease activity and prognosis (17–19), facilitating the differential diagnosis of IMN and SMN. In our literature review, only one patient was positive for the anti-PLA2R antibody, while the six patients we followed up were all negative for serum anti-PLA2R antibodies. Therefore, MN patients with a negative anti-PLA2R antibody need to be alert for the development of malignancy (20).

In recent years, a novel autoantigen of MN, THSD7A (platelet-reactive protein type 1 domain 7A), has also been identified, which accounts for 2%–3% of the prevalence of MN in adults and is the second autoantibody in MN (21). THSD7A is detected in approximately 10% of PLA2R-negative patients, and THSD7A-associated MN is associated with the development of malignancies. It has been shown that the positivity rate of THSD7A in malignant tumors is 15%–20% (22), and the messenger RNA of THSD7A was detected in some tumor tissues (gallbladder cancer) and THSD7A protein was detected in dendritic cells of tumor-infiltrating lymph node germinal centers (23, 24). One case with lung squamous cell cancer had positive THSD7A staining in both glomeruli and cancer cells. Additionally, remission of MN was observed after surgical resection of lung cancer, supporting a mechanistic role for THSD7A in the association between cancer and MN (25). In our study, two of the six patients had positive THSD7A staining in renal tissue, and one of them had double-positive staining for PLA2R and THSD7A, suggesting that patients with positive THSD7A staining were more likely to develop tumors. In other studies, THSD7A is differentially expressed in different solid tumors, including lung, breast, kidney, and colorectal cancers, which are of value for the prognosis of the disease (26). Therefore, the detection of THSD7A provides some clues for the identification of cancer-associated nephropathy.

Autoantibodies to the neural epidermal growth factor-like 1 (NELL-1) are a recent addition to the MN disease spectrum and may be more prevalent than anti-THSD7A antibodies (27). NELL-1-associated MN was identified in approximately 16% of PLA2R-negative MN cases, representing an approximate 2.5% prevalence across the entire spectrum of MN (28). There are some reports of a connection between NELL-1-associated MN and malignancy, while NELL-1 staining in renal tissue was negative in all of our six cases, so more research is needed to explore the relationship between NELL-1 and malignancy.

Previous studies have found that the remission of cancer-associated nephropathy is closely related to the effectiveness of treatment of the tumor. Lefaucheur et al. (13) reported 24 cases of cancer-associated MN, and six of 12 patients with tumor remission achieved NS remission, none of the 12 patients whose tumors did not resolve had NS remission. In this study, six patients with lung cancer had remission of MN with resection of tumor lesions and systemic chemotherapy. The above cases further illustrate that whether malignancy-associated MN can be remitted is related to the stage of tumor development and the thoroughness of treatment. If the malignant tumor is detected early and has no metastasis and is surgically removed, NS may be in complete remission. NS cannot be relieved in most cases if the tumor lesions are not resected or have metastasized. However, NS may still be relieved by effective radiotherapy and/or chemotherapy.

## Conclusions

In summary, cancer-associated MN is most common in lung cancer, and some patients with lung cancer have nephropathy as the first manifestation without lung cancer-related clinical symptoms and elevated specific tumor markers. For elderly patients with MN, especially those with negative anti-PLA2R antibodies and positive THSD7A staining by immunohistochemistry or anti-THSD7A antibodies, follow-up observation should be strengthened to be alert for tumorigenic lesions. The mechanisms involved in the etiology of MN and cancer need further study.

## Data availability statement

The original contributions presented in the study are included in the article/supplementary material. Further inquiries can be directed to the corresponding author.

## Ethics statement

The studies involving human participants were reviewed and approved by Ethics Committee of China-Japan Friendship Hospital (approval number: 2019-17-K12). The patients/participants provided their written informed consent to participate in this study.

## Author contributions

QX and WL conceptualized and designed this retrospective study. QX performed most of the statistical analyses and wrote the draft manuscript. GZ, LZ, and HG assisted with the renal pathology sectioning and staining. All authors read and approved the final manuscript.

## Funding

This work was supported by college-level projects in China-Japan Friendship Hospital (2017-2-QN-19).

## Acknowledgments

This work was supported by grants from college-level projects in China-Japan Friendship Hospital, and we thank GZ and HG for renal pathology sectioning and staining.

## Conflict of interest

The authors declare that the research was conducted in the absence of any commercial or financial relationships that could be construed as a potential conflict of interest.

## Publisher’s note

All claims expressed in this article are solely those of the authors and do not necessarily represent those of their affiliated organizations, or those of the publisher, the editors and the reviewers. Any product that may be evaluated in this article, or claim that may be made by its manufacturer, is not guaranteed or endorsed by the publisher.
